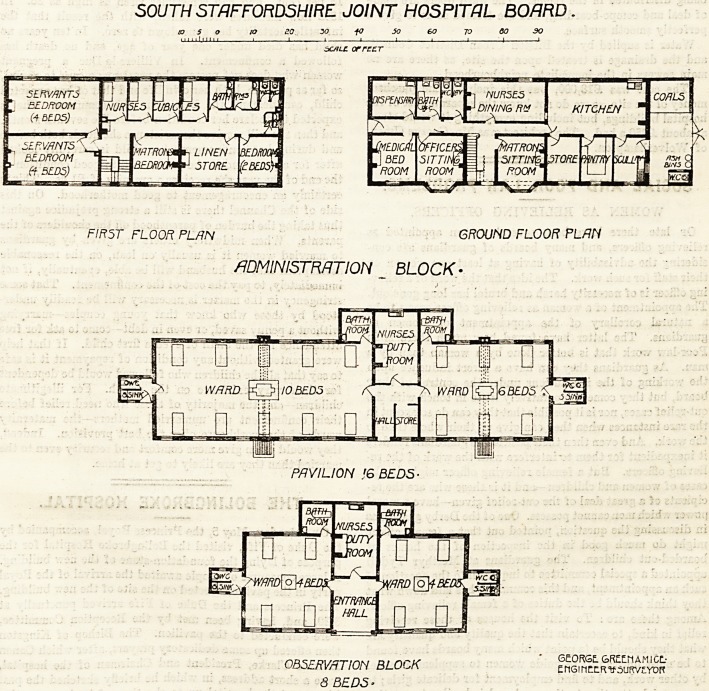# South Staffordshire Hospital for Small-Pox

**Published:** 1906-05-12

**Authors:** 


### SOUTH STAFFORDSHIRE HOSPITAL FOR SMALL-POX.

By an order of the Local Government Board the County Borough of Wolverhampton, the Borough of Smethwick, the districts of Amblecote, Bilston, Cosely, Darleston, etc., and the Rural District of Kingswinford, were formed into a United District to be known as the South Staffordshire Joint Small-pox Hospital District for the purpose of the provision, maintenance, and management of a hospital for the reception of cases of small-pox. A joint Board, under the chairmanship of Mr. Alderman Thorne, was constituted to carry the work out, and the present hospital, which was formally opened on December 5 last, is the result, and a very creditable result it is.

The population of the united district to be served by the hospital is about 440,000. A large site of 56 acres, in the Bilston District, was obtained, and of this five acres were allotted to the hospital buildings and the grounds. This is surrounded by an unclimbable fence of galvanised iron seven feet high.

The entrance is towards the north, and on the right hand near this entrance is a block which contains the porter's lodge and the discharging-rooms. Then, close by, there are two concrete bases on which additional pavilions can be erected or on which some temporary accommodation could be put up if need be. Further south is the hospital proper, which consists of four blocks, there being two pavilions, which contain sixteen beds each, an observation block with eight beds, and an administrative block. These various units are connected with each other by covered ways. The administrative block, which is a two-story building, contains rooms for the resident medical officer, the matron, fourteen nurses and domestics, kitchen, nurses' dining-room, surgery, and all other offices. This block is compact, and its component parts are conveniently arranged with reference to each other.

The pavilions are placed east and west of the administrative block, and have their long diameters running north and south. Each is divided into two wards, one containing ten beds and the other six beds. The nurses' duty-room, the store-room, the hall, and the connecting passage separate these wards from each other. A bath-room is attached to each ward, and opens directly into it. In a ward containing only ten beds, and for this special object of the treatment of small-pox, there is something to be said in favour of this plan, and perhaps the only serious objection is that one of the beds in the dormitory has a window on one side only. It is pointed out that the proximity of these bath-rooms to the ward kitchens simplifies the hot-water supply arrangements, a matter always of some moment, and one greatly to be desired when no skilled artisan is resident in or near the building. All the beds, except the one referred to, have windows on both sides, and we should like to have seen windows in the south ends of the wards also. The closets and sinks project respectively from the north and south ends of the wards, and they are properly cut off by cross-ventilated passages.

The observation block is placed between the pavilions, and faces nearly south. In design it is very similar to the other pavilions, but it contains only eight beds in two dormitories of four beds each.

Towards the east are the laundry, stables, ambulance shed, the destructor, disinfector, and mortuary.

The buildings are carried out in red brick, and the design is quite simple, as, indeed, in such hospitals it ought to be. The blocks stand on concrete bases, and these are interlaced with steel joists. The floors are of teak and the walls are faced with hard cement. There are no mouldings, or acute angles, or dust-traps of any kind. All the corners are rounded off.

The ventilation and heating are carried out by natural means. The windows are constructed with hoppers at the top and double-hung sashes at the lower part. Moorwood stoves are placed in the centres of the wards. These draw in fresh cold air, which passes through the stoves before being distributed in the rooms. The ward doors are made of deal and compo-boarding, and are framed so as to give a perfectly smooth surface.

Water is suplied by the Bilston Urban District Council, and the drainage is treated upon the site, as there are no main sewers in the immediate neighbourhood.

The cost was £18,000, but this includes the purchase money of the site, so we do not know the separate cost of the hospital buildings, but including everything it would stand at about £450 a bed. The architect was Mr. George Green, of Wolverhampton.

## Figures and Tables

**Figure f1:**